# Comparison of the Differences Between Web-Based and Traditional Questionnaire Surveys in Pediatrics: Comparative Survey Study

**DOI:** 10.2196/30861

**Published:** 2021-08-26

**Authors:** Heping Fang, Ruoling Xian, Zhuoying Ma, Mingyue Lu, Yan Hu

**Affiliations:** 1 Department of Child Health Care Children’s Hospital of Chongqing Medical University Chongqing China

**Keywords:** pediatrics, survey, questionnaire, web survey, comparative study

## Abstract

**Background:**

A web-based survey is a novel method for data capture. Some studies have applied web-based surveys in pediatrics, but few of them have reported data on the differences between web-based and traditional questionnaire surveys.

**Objective:**

The objective of our study was to evaluate the internal consistency of a web-based survey and compare it with a traditional questionnaire survey in pediatrics.

**Methods:**

A convenience sample of caregivers was invited to participate in the survey on feeding patterns and their children’s eating behaviors if their children were aged 2 to 7 years. A web-based survey and a traditional questionnaire survey were carried out between October 2018 and July 2019. A total of 1085 caregivers were involved in this study, and they were divided into the following three groups based on methods and sources: (1) web-based survey from a web source, (2) web-based survey from a hospital source, and (3) traditional questionnaire survey from a hospital source. The data were then compared and analyzed.

**Results:**

A total of 735 caregivers participated in the web-based survey and 350 caregivers participated in the traditional questionnaire survey, and 816 cases were then included in the analyses after data processing. The effective rate of the web-based survey was 70.1% (515/735), and the completeness rate of the traditional questionnaire survey was 86.0% (301/350). There were no significant differences between web-based surveys from different sources. However, demographic characteristics were significantly different between the web-based and traditional questionnaire surveys, mainly in terms of age and caregivers (*χ*²_4_=16.509, *P*=.002 and *χ*²_4_=111.464, *P*<.001, respectively). Caregivers of children aged 2 to 3 years and grandparents were more likely to respond to the web-based survey. Age-specific stratified analysis showed that the score of “monitoring” and the reporting rate of “poor appetite” in children aged 2 to 3 years were significantly higher in the web-based survey compared to the traditional questionnaire survey after adjusting for demographic characteristics.

**Conclusions:**

A web-based survey could be a feasible tool in pediatric studies. However, differences in demographic characteristics and their possible impacts on the results should be considered in the analyses.

## Introduction

A questionnaire survey is an important method in social science studies. The method was invented in the 1930s and has been widely adopted in psychological studies. With the advent of technology, a web-based survey has been appreciated as a novel method and has been applied in clinical studies [1]. To date, the number of “netizens” (citizens of the internet) in China has reached 989 million, with a 70.4% rate of internet penetration [2], which has provided an effective platform for web-based surveys. In addition, the development of mobile internet technologies has resulted in flexible web-based surveys due to mobile phone apps. The technology has a simple design and production, and has a growing frequency of usage.

Due to convenience, there has been more adoption of web-based surveys in large-scale studies. Although a larger sample size is associated with more statistical confidence of the results, this depends on the requirements and specifications of the survey design. Thus, robust study designs determine the web-based survey results, especially in the medical and health fields [3]. Although previous studies have shown that the accuracy of a web-based survey may be higher, it is primarily used in basic information collection, satisfaction evaluation, and marketing [4]. In addition, findings from fields related to subjective cognition have been shown to be highly vulnerable to survey methods [5]. In pediatrics, the participants of questionnaire surveys are mainly caregivers, who are often subjective; thus, the adopted survey methods might yield varying data. While some studies have applied web-based surveys in pediatrics, data on the differences between web-based and traditional questionnaire surveys remain scant. Here, we evaluated the internal consistency of a web-based survey and then compared it with a traditional questionnaire survey in pediatrics.

## Methods

### Study Design

This study was conducted between October 2018 and July 2019. We used two survey methods to investigate eating behaviors in children aged 2 to 7 years, as well as the feeding patterns of their caregivers. We conducted a web-based survey in October 2018, while a traditional questionnaire survey was conducted in July 2019. Based on the methods and sources, the participants were divided into the following three groups: (1) web-based survey from a web source (group A), (2) web-based survey from a hospital source (group B), and (3) traditional questionnaire survey from a hospital source (group C).

The study was reviewed and approved by the Ethics Committee of the Children’s Hospital of Chongqing Medical University (2019-202).

### Traditional Questionnaire Survey

The traditional questionnaire survey was mainly conducted by the investigators through face-to-face questioning in the child health care clinic, and informed consent was obtained orally before the investigation. The completed questionnaires were then collected and kept by the investigators. The questionnaire (43 items) included basic information about the children and the caregivers, and required the caregivers to evaluate the children’s performance in the previous month using the Likert scoring method [6].

The simplified Child Feeding Questionnaire (CFQ) revised by Lixia et al [7] was used to assess the feeding patterns of the caregivers. The content included “monitoring,” “pressure,” and “restriction,” each of which involved four items, making a total of 12 items. The score for each dimension was expressed as an average score for the four items in that dimension.

On the other hand, the simplified Chinese version of Identification and Management of Feeding Difficulties (IMFeD) [8] was used to investigate the children’s eating behaviors. The content included five aspects of eating behavior problems, including “poor appetite” (four items), “food preference” (three items), “poor eating habit” (four items), “parental misperception” (two items), and “fear of feeding” (two items). An eating behavior problem existed when the score was lower than 40% of the total in each aspect.

### Web-Based Survey

The web-based survey was set as an open survey. It was built by “wjx” (an online questionnaire platform in mainland China) and was promoted remotely through WeChat on the internet (web source), as well as a paper poster (Multimedia Appendix 1) with a QR code in the child health care clinic (hospital source). Initial contact with potential participants was made on the internet, and the questionnaire could be accessed directly through WeChat. The questionnaire contained five pages, and a brief introduction of the questionnaire was shown on the first page. The main content and scoring methods for the web-based survey were similar to the traditional survey, and basic information was collected by adaptive questioning. There were, however, three additional items in the web-based survey that were used to distinguish between (1) the web and hospital source, (2) healthy and sick children, and (3) high and poor quality of the response (self-assessment), resulting in a total of 46 items. The survey time was set at about 10 minutes. To ensure the honesty of the participants, survey participation was anonymous and voluntary. To ensure participation, feedback and suggestions would be shared at the end of the survey. To reduce repeated responses, each WeChat account of the survey could only send a single response. In addition, to reduce the possibility of misunderstanding, 10 caregivers were invited to adjust the expressions through pretests prior to the survey. Finally, the participants were required to complete all items before submitting the survey, and the answers could not be reviewed or changed.

### Data Processing

Only investigators had access to the data. Data, such as IP address, phone number, and children’s and caregivers’ basic information, were recorded in Excel 2016 (Microsoft Corp). Then, the data were checked for duplication, and cases with a high degree of overlap (if any of the three aspects mentioned above were the same) were considered as repeated responses. Because psychological theory appreciates the first impression as more accurate [9], late responses, which were repetitive, were excluded. Finally, cases of sick children (eg, chronic liver or kidney diseases, developmental diseases, and food allergy) and poor quality of responses (ie, the participants themselves thought the answers were not rigorous) in the web-based survey were also excluded from the analyses.

### Statistical Analyses

The sample in this study was a convenience sample, and the sample size was calculated using a single proportion sample size estimating algorithm [10]. An 82.8% rate of incidence of eating behavior problems was taken from our previous study [11], with a margin of error of 5%, confidence level of 95%, and nonresponse rate of 10%. Calculation provided 217 as the minimum sample size for each group.

Only completed questionnaires were analyzed. All statistical analyses were performed using SPSS 26.0 (IBM Corp). Descriptive statistical tools were used to analyze the demographic characteristics of the participants. To test whether the children from the hospital sources (groups B and C) had nutrition-related problems, comparison between BMI-for-age Z scores and the standard mean was carried out using a one-sample *t* test. We used analysis of variance (ANOVA) and the least significant difference (LSD) test to compare the feeding patterns. On the other hand, comparison of eating behavior problems was carried out using the chi-square test. Bonferroni correction was utilized for multiple comparisons. A *P* value <.05 was considered statistically significant.

## Results

### Data Processing and Response Rates

**Figure 1:**
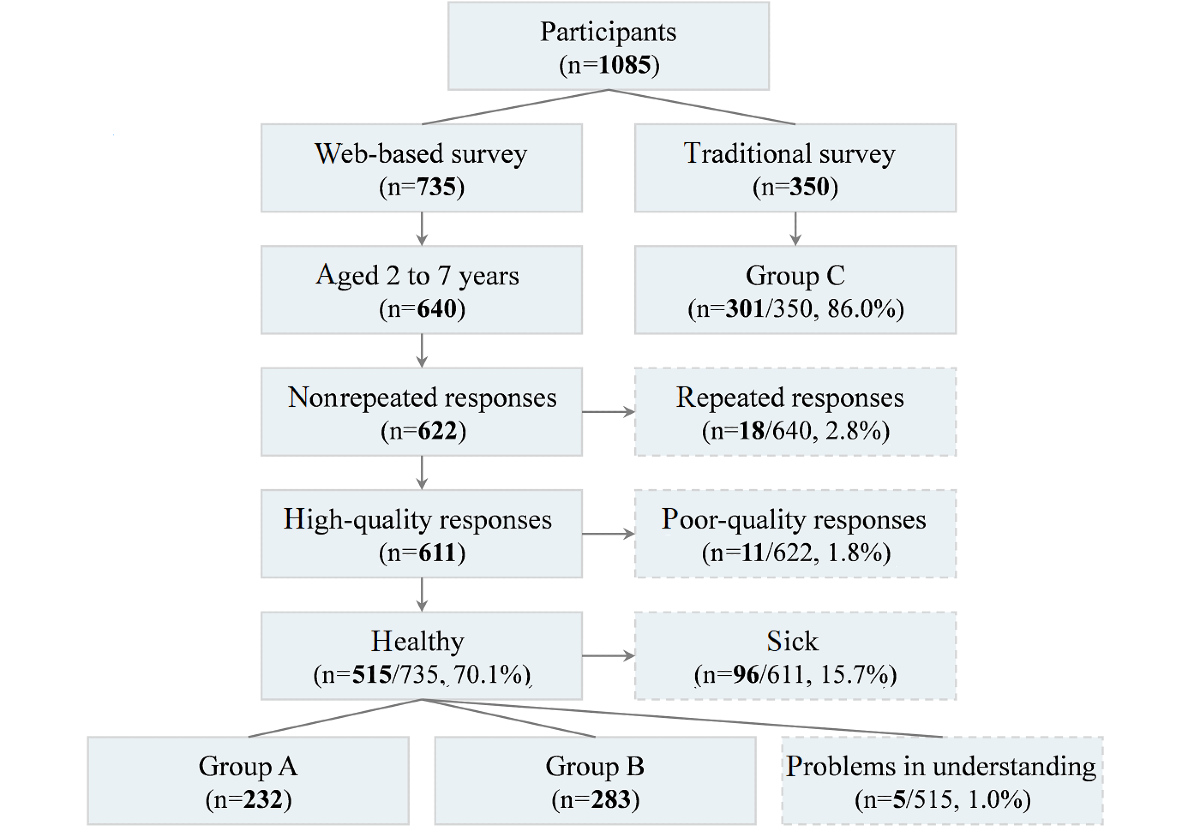
Data processing and the response rates of the surveys.

Data processing and response rates are shown in Figure 1. A total of 1085 caregivers (735 were in the web-based survey and 350 were in the traditional questionnaire survey) were involved in this study. It took 9.9 (IQR 4.9) minutes for the web-based survey and about 10 minutes for the traditional questionnaire survey. There were 232 cases in group A, 283 in group B, and 301 in group C after data processing. The effective rate of the web-based survey was 70.1% (515/735), and the completeness rate of the traditional questionnaire survey was 86.0% (301/350).

### Characteristics of the Participants

Our data showed that the mean BMI-for-age Z scores in groups B and C were −0.06 (SD 1.08) and −0.09 (SD 1.31), respectively, which had no significant differences with the standard mean (*t*_237_=−0.890, *P*=.37 and t_271_=−1.133, *P*=.26, respectively). Thus, the children from hospital sources had no nutrition-related problems.

The chi-square test showed significant differences in age and caregivers among the three groups (*χ*²_4_=16.509, *P*=.002 and *χ*²_4_=111.464, *P*<.001, respectively). In addition, multiple comparisons showed that there were no significant differences between groups A and B. On the contrary, the results showed that there were significant differences between groups A and C in age and caregivers (*χ*²_2_=14.218, *P*=.001 and *χ*²_2_=103.387, *P*<.001, respectively), as well as between groups B and C in age and caregivers (*χ*²_2_=9.359, *P*=.009 and *χ*²_2_=96.940, *P*<.001, respectively). The findings showed that caregivers of children aged 2 to 3 years and grandparents were more likely to respond to the web-based survey (Table 1).

**Table 1:** Comparison of demographic characteristics among the three groups.

Characteristic			Group A^a^ (N=232), n (%)		Group B^b^ (N=283), n (%)		Group C^c^ (N=301), n (%)		*χ*^2^ (*df*)^d^		*P* value
**Age**									16.509 (4)		.002
	2-3 years	106 (45.7%)		117 (41.4%)		90 (29.9%)^e,f^					
	3-5 years	87 (37.5%)		119 (42.0%)		141 (46.8%)					
	5-7 years	39 (16.8%)		47 (16.6%)		70 (23.3%)					
**Gender**									1.297 (2)		.52
	Male	122 (52.6%)		163 (57.6%)		167 (55.5%)					
	Female	110 (47.4%)		120 (42.4%)		134 (44.5%)					
**Caregivers**									111.464 (4)		<.001
	Parents	146 (62.9%)		185 (65.4%)		290 (96.3%)^e,f^					
	Grandparents	79 (34.1%)		90 (31.8%)		6 (2.0%)					
	Others	7 (3.0%)		8 (2.8%)		5 (1.7%)					

^a^Group A: web-based survey from a web source.

^b^Group B: web-based survey from a hospital source.

^c^Group C: traditional questionnaire survey from a hospital source.

^d^Chi-square analysis with Bonferroni correction.

^e^*P*<.01 vs group A.

^f^*P*<.01 vs group B.

### Feeding Patterns and Eating Behavior Problems

Age-specific stratified analysis was performed following selection of children whose caregivers were parents to avoid the influence of demographic characteristics on the data. The results showed that in children aged 2 to 3 years, the scores on “monitoring” and the rates of reporting “poor appetite” were significantly different among the three groups (*F*_2_=12.549, *P*<.001 and *χ*²_2_=6.579, *P*=.04, respectively). Multiple comparisons then showed that the scores on “monitoring” in groups A and B were significantly higher compared to that in group C (*P*<.001 and *P*<.001, respectively), while the rate of reporting “poor appetite” in group B was significantly higher than that in group C (*χ*²_1_=6.138, *P*=.01). In children aged 3 to 5 years, the scores on “restriction” were significantly different among the three groups (*F*_2_=3.221, *P*=.04), but multiple comparisons showed no significant differences between the groups. In children aged 5 to 7 years, the rates of reporting “poor appetite” were significantly different among the three groups (*χ*²_2_=6.472, *P*=.04), but multiple comparisons showed no significant differences between the groups.

## Discussion

### Principal Findings

Compared with a traditional questionnaire survey, a web-based survey is widely accepted due to its convenience, low cost, efficiency, wide-range coverage, and semiclosed and anonymous nature. However, it presents other shortcomings, such as repeated responses as well as information and selection bias, which might significantly affect the results of descriptive studies [12-14]. It is, therefore, necessary to conduct appropriate pretests [15] and choose reliable scientific data processing methods before executing a web-based survey. In our study, a variety of methods, such as pretests and outcome feedback, were adopted to improve the robustness of the web-based survey. Our results showed that only 2.8% (18/640) of the cases were classified as repeated responses, while 1.8% (11/622) were of poor quality. Moreover, the effective rate of the web-based survey was 70.1% (515/735), which was lower than the completeness rate of the traditional questionnaire survey (86.0%, 301/350). This is because the web-based survey was set as an open and voluntary survey, resulting in 95 participants (12.9%, 95/735) who were not study participants. These findings suggest that a web-based survey could be a feasible tool in pediatrics. Most importantly, the different data sources did not impact the output of the web-based survey, indicating that the web-based survey had good internal consistency whether through web promotion or hospital publicity. However, despite revising the questions based on the pretests, 1.0% (5/515) of the cases reported difficulty with understanding the content. Thus, lack of investigator explanation might lead to confusion of the results due to misunderstanding among participants.

We next assessed whether the different sources of data could impact the results. Benedik et al [16] showed that both web-based and traditional questionnaire surveys demonstrated no differences in nutrient intake among pregnant women. Although the study ensured the consistency of participants, highly educated participants may make the conclusion unrepresentative. In addition, since nutrient intake represents objective data, it may not be easily affected by the survey methods. On the contrary, Milton et al [17] showed that the web-based survey had a higher reporting rate on sensitive topics in young people, suggesting that the results between the two methods of surveys might differ even in cases where the participants are the same. The effects of web-based and traditional survey methods among different populations have also been evaluated. Graefe et al [18] reported different demographic characteristics between web-based and mail-based surveys, but the participants of the web-based survey were younger and had better education. In our study, caregivers of children aged 2 to 3 years and grandparents were more likely to participate in the web-based survey, which is different from the finding of other studies [18,19] reporting that older people are less likely to participate in a web-based survey. This may be explained by the fact that today’s grandparents are not exactly the same as older people, and the widespread popularity of the internet in China [2] has undoubtedly changed how people use mobile phones. More importantly, different demographic characteristics might lead to different results. For instance, Cantuaria et al [19] demonstrated that participants in a mail-based survey were more likely to report health problems. In our study, we showed that not only the eating behaviors of children but also the feeding patterns of caregivers were inconsistent, even after adjusting for demographic characteristics, a phenomenon dominant in the younger age group. We associated these findings with the fact that our survey focused on children’s behavioral development, and the answers were more subjective and more dependent on the feelings of the parents. Thus, a web-based survey might be more prone to information bias in pediatrics when it comes to subjective topics. Besides, the interpretations of the investigators might play a certain role in the traditional questionnaire survey and the psychological effects of the participants may influence them to give a “good” answer [20]. In addition, although the web-based survey was meant to be a self-evaluation model to improve participation, the potential selection effect of its voluntary nature on participants could not be completely ruled out, which might lead to an increased proportion of problematic participants. Taken together, our data suggest that demographic characteristics and results would be different among different survey methods when used in pediatrics, and topics with strong subjectivity need more comprehensive consideration before adopting the study tools.

### Limitations

Our study was limited by the convenience sample, as well as the fact that the interval between the two surveys was slightly long and the participants were not the same. Although stratified analysis was conducted according to demographic characteristics, changes in the feeding and eating behaviors of the participants and their effects on the results cannot be completely ruled out.

### Conclusions

Our data demonstrated that a web-based survey could be a feasible tool in pediatrics. However, differences in demographic characteristics and their possible impacts on the results should be considered when interpreting data from a web-based survey.

## Appendix

Multimedia Appendix 1 The paper poster used in the survey.
