# Supplementary figures and images for: Comparison of the Differences Between Web-Based and Traditional Questionnaire Surveys in Pediatrics: Comparative Survey Study
Source: J Med Internet Res. 2021 Aug 26;23(8):e30861. doi: 10.2196/30861 (PMC8430848; doi:10.2196/30861)

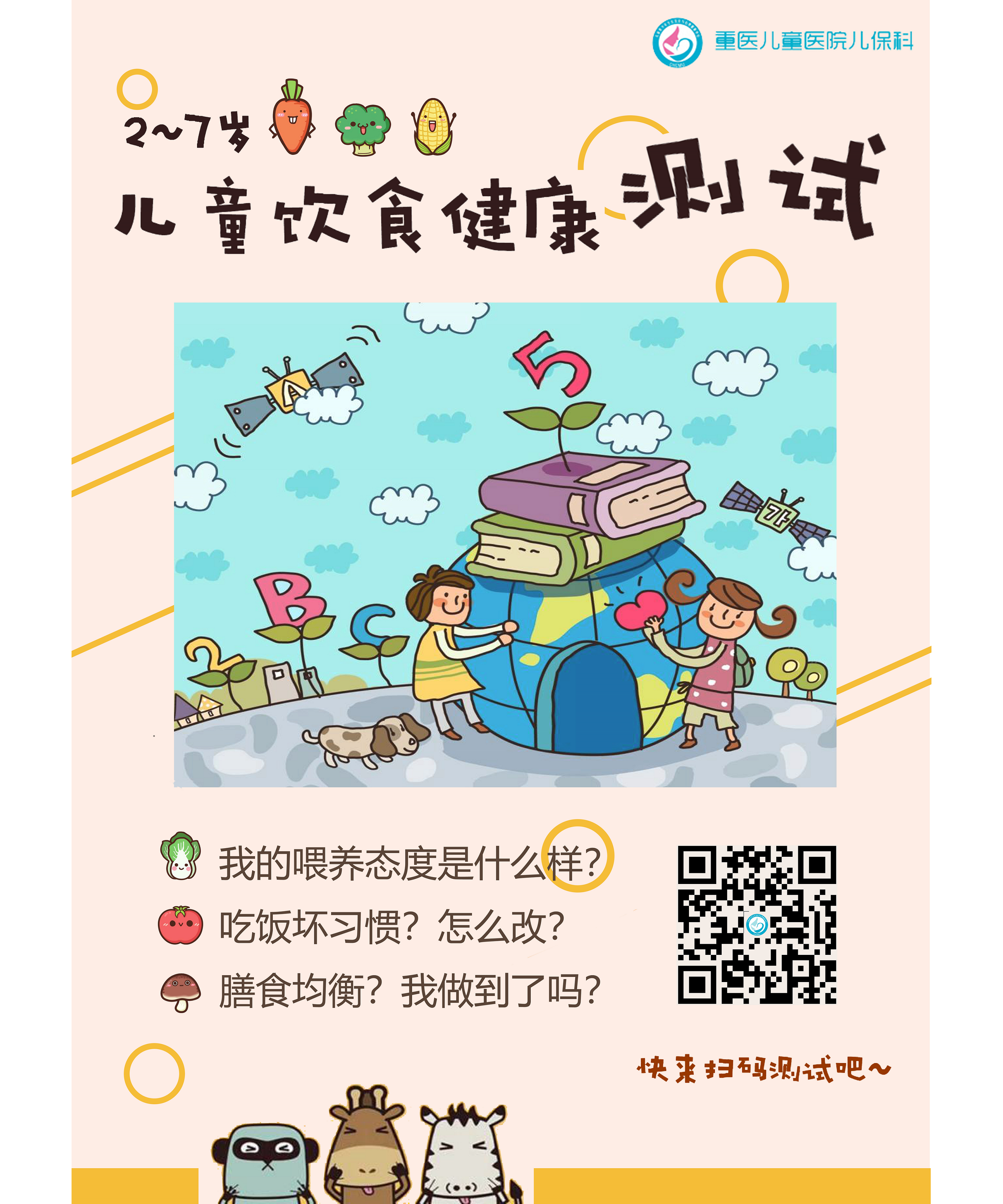

Supplement: Multimedia Appendix 1 [file jmir_v23i8e30861_app1.png]
